# Biostatistical and medical statistics graduate education

**DOI:** 10.1186/1472-6920-14-18

**Published:** 2014-01-28

**Authors:** Michael B Brimacombe

**Affiliations:** 1 Department of Biostatistics, KUMC, Kansas City, USA

## Abstract

The development of graduate education in biostatistics and medical statistics is discussed in the context of training within a medical center setting. The need for medical researchers to employ a wide variety of statistical designs in clinical, genetic, basic science and translational settings justifies the ongoing integration of biostatistical training into medical center educational settings and informs its content. The integration of large data issues are a challenge.

## Biostatistical and medical statistics graduate education

The quantitative education of graduate students in the health sciences is an important area of consideration in a modern internet based world. The onset of massive datasets and so-called Data Science and the related growth of computer science and engineering based programs in this area [1], has implied a need to carefully delineate and justify health-related statistical and biostatistical training, its core elements and how it may incorporate aspects of BigData approaches to the analysis of data structures in the health sciences. Such approaches typically reflect the massive nature of many health related databases and clinical data repositories where standard statistical methods may not easily apply. Even practical issues such as creating data architectures and ontologies that can be searched and subject to statistical analysis are challenging [1].

More generally, understanding how the concepts of randomness apply to the design and interpretation of clinical trials, observational medical studies and the improvement of clinical practice generally is of relevance to a wide audience of healthcare students and professionals [2]. Here we focus on graduate level training for students in the area of biostatistics in a medical center or school of public health environment, where the learning of statistical theory must be balanced with a wide variety of health related applications.

### Definition

Statistics and biostatistics are areas of applied mathematics that are required for the proper design and analysis of most experimental or observational data. They require an understanding of probability, related statistical models and theories of inferential probability. These areas are somewhat unique as their progenitors (Fisher [3], Pearson [4], and Jeffreys [5]) were mostly applied scientists who wrestled with the need to interpret real world experiments and data in the context of scientifically rooted mathematical or probability models, with applications including early population genetics, time series analysis, biometry and toxicology.

Biostatistics is a discipline with a long history and wide area of application, often termed biometrics in its early days, a word that has now taken on other meanings, it examines the application of statistical theory to biological problems of various types. With the onset of clinical trials and pharmaceutical studies, it has developed in the directions of clinical trial methodology, health outcomes research, survival analysis, longitudinal models and generalized linear models of various types [2,6].

The role of probability in inference and (clinical) decision making is now well established [7]. The importance of incorporating randomly occurring variation when interpreting clinical results justifies the application of statistical techniques in a wide variety of medical and health related decision making. Elements of randomness also underlie most experimental settings and the interpretation of the many physiologic and biochemical measurements. At a minimum the understanding of p-values, confidence intervals, t-tests, ANOVA tables and basic statistical models such as linear or logistic regression, are an important aspect of the quantitative literacy that medical professionals, clinical researchers and basic scientists should all possess to interpret medical research findings [2]. The related area of Bayesian statistics with subjective risk and decision based clinical assessment is also a growing subject requiring appropriate integration into quantitative curriculums [8].

### Where does it live

Historically biostatistical training has occurred in mathematics departments, with the hope that students can develop the ability to collaborate or consult with scientists. Over time this training has begun to migrate in some settings to medical centers and biostatistics departments in medical center settings, where faculty and the offered training exist in a medically oriented research setting. This results in a slightly less mathematically oriented curriculum, but a more focused set of priorities, often emphasizing collaboration with basic scientists and clinicians.

Schools of public health have also become home to biostatistical programs [9]. Here the focus is often on larger clinical trials and large healthcare database settings, different from the laboratory or clinical trials related studies that often predominated in medical centers. Biostatistics as a component of a medical center setting with or without a school of public health reflects a web of inter-related relationships and opportunities; supporting cancer center clinical and genetic research, nursing related health outcomes research, basic science research and clinical trials oriented research. This provides many opportunities for real world application as an essential component of biostatistics training. Often the responsibility for dealing with large scale data management falls to biostatisticians in addition to more traditional research efforts, though the growth of medical informatics is creating a need for informatics specialists who may or may not utilize statistical analysis in their work, focusing instead on Data Science and empirical models [10].

The overlap with medical or clinical settings may also draw biostatisticians into the world of training M.D.s, clinicians and related health professionals active in clinical medicine. In this setting, biostatistician groups or departments may offer certificates or degrees in aspects of clinical research for clinicians seeking to engage in research. This is an area where determining the degree of quantitative literacy M.D.s active in research should possess is open to further development [11]. Often these settings have a large online component to them and the challenge of teaching statistics online to medical students arises [12].

## Challenge: big data and data science

A challenge to biostatistics programs and all statistical programs is how to incorporate, interact with the phenomenon of BigData. Data science as it is being called is mostly the handling of massive datasets with multiple strata and thousands of variables. There is often limited statistical input apart from many different types of clustering algorithm or multiple association programs. Clinical data repositories and massive hospital data systems are becoming common and viewed as a potential tool for healthcare quality assessment, clinical trials and health outcomes research. Under the name of Informatics such computer science and engineering driven approaches are drawing the attention of the National Institutes of Health, creating massive clinical databases. However visualizing the data in such settings is a challenge, much less applying standard statistical methodology (standard errors and p-values) that have may have little or no meaning in very large sample sizes.

As this new science finds its home, perhaps on the engineering and computer science side of the ledger, it will be a challenge to biostatisticians to remain involved in the development of software and methodology in this area. This will impact support for training and the practical development and growth of biostatistical programs. Much of this future is currently being decided [1] and often in the medical school or center setting where cost is a concern vis-a-vis output of research results.

## Teaching philosophy & curriculum

Biostatistics, at its core, is a mathematical subject, with its own set of core ideas, concepts and controversies. It typically differs from mathematical statistics training by incorporating more medically oriented statistical techniques and applications, and less theoretical probability theory. The teaching of a mathematical subject is by definition a challenge. Principles and mathematically justified procedures must be clearly and carefully defined and explained. Problems are often proof-based in advanced theory courses and require detailed applications in applied courses. Exposure to advanced computational skills and methods are also expected along the way. As retention is a goal, especially at the advanced levels, where ultimately a thesis must be produced, didactic teaching and mentoring is often emphasized. This may be at odds with the push to online in much of medical training.

The biostatistics curriculum in particular is a mixture of theory and application, reflecting the reality of modeling biological and medical phenomenon. This includes the analysis of real world datasets, which makes biostatistics and statistics somewhat unique in the mathematical world. The fitting of theorized mathematical models to data and the use of the resulting expected properties of these models for inference is the central challenge of much biostatistics. This requires exposure on the part of the student to consulting and collaboration, often with clinicians and basic scientists. This type of mentoring is a challenge but necessary to produce a quality biostatistician/researcher at the end of their studies.

The teaching of mathematics has a long and studied history. These tend to focus on basic concepts and mostly undergraduate education [13]. The philosophy of teaching statistics reflects similar considerations [14], and the evaluation of various approaches to the teaching of statistics is an ongoing effort [15,16]. Given the wide variety of applications for which biostatisticians must train, if the student does not have a strong mathematical background, they may struggle with coursework and the writing of a thesis, but if they lack experience with health related applications, they may also face difficulties with the consulting aspect of the training process.

Given the cutting edge nature of much applied biostatistical work, there is ongoing discussion regarding the intellectual content of the biostatistics curriculum. This typically includes; modern statistical computing, frequentist methods, nonparametrics, likelihood and Bayesian approaches, mathematical statistics, design of experiments, linear models, survival analysis, longitudinal analysis, latent variable models, nonlinear models, categorical data analysis and clinical trials. See Table 1.

**Table 1:** List of typical core PhD, electives and future choices

**Core Ph.D.**	**Electives**	**Future**
Theoretical statistics	Advanced epidemiology	Visualization of large databases
Survival analysis	Analysis of healthcare data	Data algorithm development
Categorical analysis	Introduction to genomics	Computational platforms and structures
Longitudinal analysis	Aspects of U.S. medicare data	Large database inference
Structural equations models	U.S. Veterans Affairs data analysis	Programming big data
Multivariate analysis	Image analysis	Genetic function and structure
Bayesian analysis	Advanced programming	Analysis of restricted models

Such a mixture of theory, practice and applied modeling oriented courses allows the student to blend the designing of mathematical models and their underlying optimality properties with the fitting of these models to biomedical data. Interpreting the actual import of the fitted models is often a joint exercise with both biostatistician and clinician/researcher discussing the relevance and importance of the results. In the context of public health for example, the need to be fluent in various statistical methodologies and knowledgeable in applications to health policy, epidemiology and other public health areas is important.

The development of electives, primarily related to the application of statistical and informatic methods in related fields is important to broaden and fill out the education of future biostatistians. Epidemiology, disease processes, genomics, large data handling and investigation, informatics, health economics and outcomes research are all possible electives as are aspects of genetics. In reality, access to these courses may be limited by resources, agreements between school and departments and must be carefully organized. The teaching loads attached to the rather conservative listing given in Table 2 is a serious challenge in smaller departments.

**Table 2:** Areas and types of applications

**Large data**	**Public health/health economics and policy**	**Medicine**	**Future**
Genomics	Smoking cessation	Diabetes	Illness behavioral profiles
Brain imaging	Prevention	Neurological conditions	Health data mining
Medicare data	Utilization	Pallative care	Cost patterns identification
Clinical repository development	Health economics	Fetal and newborn care	Rare disorder identification

Give the polymath nature of many biostatistical positions and the mostly underappreciated ability of statisticians to modify the statistical design template for each applied setting; it is very useful for students to become fluent in the qualitative aspects of their thesis research. Students with knowledge of an applied area can more easily function as a collaborator and build a future career. The “generalist” label is not generally respected in research settings. Required and essential, defining of many biostatistical positions, but often not respected.

## Learning centers

It is worth considering the various learning formats available in a medical center setting in relation to biostatistics. Medical training has to a large extent moved to an online platform, with specific goals and a very parsed approach to the dissemination of knowledge. At the undergraduate level, the courses are very focused on aspects of anatomy, physiology, biochemistry etc. and the goal is to both succeed at the course level and the medical board exams. As such exposure to statistics or epidemiology tends to be in the form of brief three-week intensive courses with the learning geared to success on the exams. Research level biostatistics may become relevant in the last medical school year or at the resident/fellow stage.

In this context, biostatistical courses, often in standard fourteen week formats, are a separate entity, focused on M.S. and Ph.D. level training, often viewed as old-fashioned given the need to teach higher level mathematics at a pace which allows students to grasp, digest and apply theoretical concepts. Allied health, health policy and nursing typically also provide Ph.D. level training and as such will tend to have similar didactic teaching formats in the medical center context. The need to be aware of this diversity and integrate the different teaching cultures is a necessity in a medical center environment where the end product is well trained collaborative science, scientists and funded research.

## Online issues

Much teaching in medical school and health related settings has migrated to online settings where the material presented to students can be standardized and presented in an easily updated format [17,18]. While this may be appropriate for information and techniques relevant to medical training, the teaching of higher-level mathematical and statistical ideas in online formats is very new and remains an area of ongoing assessment [16,19]. When applications are the core of the material, the online setting can be very useful, but if methods themselves are to be taught, including their theoretical justifications, the use of online settings remains an open issue. Much of the usefulness and relevance of online formats, especially in regard to retention, graduation rates and the quality of the graduates, remains a question for study [17-19]. While the technology is new and impressive, the ability of students to absorb mathematically related ideas from rather restrictive online settings seems to be successful for students who can learn independently, a path that becomes more difficult as the student moves to higher level courses [16].

The standardization of teaching imposed on much of the medical curriculum may not be a template for the presentation and learning of mathematical and statistical ideas. The development of online settings specifically tailored for these ideas in medical settings is very much a work in progress [12,19]. Extending these into settings directly relevant to the statistical aspects of medical research is an ongoing challenge. Assessment and longer term follow-up will be required as online platforms become more prevalent.

## Health research and statistical ideas

The defining of new statistical ideas and their applications is a key aspect of Ph.D. training. In biostatistics, the development and investigation of specific types of models and their properties tends to support most research in the setting of graduate programs. For example categorical models, longitudinal models, survival models have been the subject of a very large number of dissertation projects over the past thirty years.

However, looking to the future, the new world of massive data, some of it clinical or genetic in nature, is certainly imposing and growing in importance as a challenge to biostatistical methodology, very little of which was developed for such large settings. Questions such as “Is the current level of training in this area appropriate?” “Can the world of informatics be left to non-statisticians?” seem very relevant.

Genomics and the search for genetic structures within large genomic databases have emerged as a component of training. Bioinformatics is often viewed as a separate entity, and much of it is not directly statistical in nature, as the issue of how to process such large datasets is a dominating consideration. Public health also requires the analysis of large healthcare related databases, both specifically and in relation to issues affecting the ongoing restructuring of the U.S. health system and is also a key area of potential research for biostatistics. BigData issues arise throughout here and many standard statistical methods perform poorly if analyses are to be conducted on millions of health care records.

In deciding what is a potentially important area of future research, it helps to consider where biostatisticians will fit in future research endeavors in both academia and industry. Are biostatisticians to form a bridge among the various applied disciplines, functioning as quantitative and design oriented collaborators with their own areas of application, or are they to function as technicians supporting clinicians and basic scientists including geneticists at a high level of application. Biostatisticians tend to have success as lead applied researchers in secondary data analysis of for example Veterans Affairs or Census based health outcomes research, but this requires the biostatistician be very knowledgeable in the content area. This is also true in public health settings. Professors looking to guide students in selecting topics relevant to the future of statistical analysis may consider these issues, especially in relation to the interests and abilities of the individual student. Areas of research that provide the most supportive environment for a research or applied statistical career should be identified and emphasized, a challenge as these change over time. This should be considered both internally to biostatistical research and in relation to overall health research related funding.

## Support overview

It is necessary to discuss support and type of support when taking on M.S. or in particular Ph.D. students for a four-to-five year period of training. The past may not be a useful guide to the present and future. Training grants as may become more relevant to the funding of students, rather than incorporating student support directly into research grants to individual investigators. Quantitative methods may require general training, but they may also come to be seen as essential to the basic or clinical sciences, requiring specific training outside of the standard biostatistical context.

Timelines are an issue as they affect and guide the level and length of support that can be given to each student in pursuit of the degree. Most programs have a maximum of seven years, but funding typically runs to a maximum of four or five years. Programs that develop students will have to plan for longer time frames as the definition and toolkit that defines biostatistics broadens and includes more coursework and consulting experience. Biostatistical students, once at a certain level of expertise, can function directly as biostatistical support on many applied projects and funding of biostatistical training as research assistant/consultant support to collaborative projects is a possibility. This is certainly true in medical center environments, which often provide many such opportunities, though sometimes have barriers of various sorts to optimal levels of collaboration [20].

## Summary

Development of an academic and intellectual setting where students can learn and further develop advanced biostatistical methods and applied applications, is the ongoing challenge of most biostatistical graduate programs, especially in the medical center environment. In these settings excellent opportunities abound for collaboration and exposure to cutting edge research. But much concern is focused on deteriorating funding levels and a lack of experience with data driven science. There is also the related issue of being available to train medical students and clinical researchers in a variety of statistical approaches. Online platforms are common in general medical education, however remain a challenge in relation to more mathematically oriented material. The final product of meeting and integrating these various challenges will be the development of the next generation of biostatistical scientists in many of the new scientific areas of health and medical research and the place of department of biostatistics in the medical center setting.

## Competing interests

The author declares that he has no competing interests.

## Pre-publication history

The pre-publication history for this paper can be accessed here:

http://www.biomedcentral.com/1472-6920/14/18/prepub
